# Supplementary material for: HIVprotI: an integrated web based platform for prediction and design of HIV proteins inhibitors
Source: J Cheminform. 2018 Mar 9;10:12. doi: 10.1186/s13321-018-0266-y (PMC5845081; doi:10.1186/s13321-018-0266-y)
Supplement: Supplementary file 2 — Additional file 2. Source code of HIVProtI web server. [file 13321_2018_266_MOESM2_ESM.zip › HIVprotI_Source-code/hivprotI/weka-3-6-6/doc/weka/classifiers/trees/adtree/ReferenceInstances.html]

ReferenceInstances


---


|  |  |  |
| --- | --- | --- |
| **Overview** \| **Package** \| **Class** \| **Tree** \| **Deprecated** \| **Index** \| **Help** \| **Weka's home** | |  |
| **PREV CLASS**   **NEXT CLASS** | **FRAMES**    **NO FRAMES**     **All Classes** |  |
| SUMMARY: NESTED \| FIELD \| CONSTR \| METHOD | DETAIL: FIELD \| CONSTR \| METHOD |  |


---


## weka.classifiers.trees.adtree Class ReferenceInstances

```
java.lang.Object
  weka.core.Instances
      weka.classifiers.trees.adtree.ReferenceInstances
```

**All Implemented Interfaces:**: java.io.Serializable, RevisionHandler

---

``` public class ReferenceInstances extends Instances ```

Simple class that extends the Instances class making it possible to create
subsets of instances that reference their source set. Is used by ADTree to
make reweighting of instances easy to manage.

**Version:**
:   $Revision: 1.5 $

**Author:**
:   Richard Kirkby (rkirkby@cs.waikato.ac.nz)

**See Also:**: Serialized Form

---

**Field Summary**

**Fields inherited from class weka.core.Instances**

`ARFF_DATA, ARFF_RELATION, FILE_EXTENSION, SERIALIZED_OBJ_FILE_EXTENSION`


**Constructor Summary**

`ReferenceInstances(Instances dataset,
int capacity)`
  
          Creates an empty set of instances.


| **Method Summary** | |
| --- | --- |
| `void` | `addReference(Instance instance)`             Adds one instance reference to the end of the set. |
| `java.lang.String` | `getRevision()`             Returns the revision string. |

**Methods inherited from class weka.core.Instances**

`add, attribute, attribute, attributeStats, attributeToDoubleArray, checkForAttributeType, checkForStringAttributes, checkInstance, classAttribute, classIndex, compactify, delete, delete, deleteAttributeAt, deleteAttributeType, deleteStringAttributes, deleteWithMissing, deleteWithMissing, deleteWithMissingClass, enumerateAttributes, enumerateInstances, equalHeaders, firstInstance, getRandomNumberGenerator, insertAttributeAt, instance, kthSmallestValue, kthSmallestValue, lastInstance, main, meanOrMode, meanOrMode, mergeInstances, numAttributes, numClasses, numDistinctValues, numDistinctValues, numInstances, randomize, readInstance, relationName, renameAttribute, renameAttribute, renameAttributeValue, renameAttributeValue, resample, resampleWithWeights, resampleWithWeights, setClass, setClassIndex, setRelationName, sort, sort, stratify, stringFreeStructure, sumOfWeights, swap, test, testCV, toString, toSummaryString, trainCV, trainCV, variance, variance`

**Methods inherited from class java.lang.Object**

`equals, getClass, hashCode, notify, notifyAll, wait, wait, wait`

**Constructor Detail**

### ReferenceInstances

```
public ReferenceInstances(Instances dataset,
                          int capacity)
```

:   Creates an empty set of instances.

    **Parameters:**: `dataset` - the instances to get the header information from: `capacity` - the initial storage capacity of the set


**Method Detail**

### addReference

```
public final void addReference(Instance instance)
```

:   Adds one instance reference to the end of the set.
    Does not copy instance before it is added. Increases the
    size of the dataset if it is not large enough. Does not
    check if the instance is compatible with the dataset.

    :   **Parameters:**: `instance` - the instance to be added

---


### getRevision

```
public java.lang.String getRevision()
```

:   Returns the revision string.

    :   **Specified by:**: `getRevision` in interface `RevisionHandler` **Overrides:**: `getRevision` in class `Instances`
    :   **Returns:**: the revision


---


|  |  |  |
| --- | --- | --- |
| **Overview** \| **Package** \| **Class** \| **Tree** \| **Deprecated** \| **Index** \| **Help** \| **Weka's home** | |  |
| **PREV CLASS**   **NEXT CLASS** | **FRAMES**    **NO FRAMES**     **All Classes** |  |
| SUMMARY: NESTED \| FIELD \| CONSTR \| METHOD | DETAIL: FIELD \| CONSTR \| METHOD |  |


---
